# Supplementary material for: Eukaryotic Initiation Factor 2α Kinases Regulate Virulence Functions, Stage Conversion, and the Stress Response in Entamoeba invadens
Source: mSphere. 2022 May 31;7(3):e00131-22. doi: 10.1128/msphere.00131-22 (PMC9241534; doi:10.1128/msphere.00131-22)
Supplement: TABLE S1 [file msphere.00131-22-s0001.docx]

|  | **% SIMILARITY** | | | | | | | | | | | | | | | | | | | | | | | | | | |
| --- | --- | --- | --- | --- | --- | --- | --- | --- | --- | --- | --- | --- | --- | --- | --- | --- | --- | --- | --- | --- | --- | --- | --- | --- | --- | --- | --- |
| **% IDENTITY** | **KINASE**  **DOMAIN*** | **Ei_IF2K-A** | **Ei_IF2K-B** | **Eh_IF2K-A** | **Eh_IF2K-B** | **Ac_GCN2-like** | **Dd_iFKA** | **Dd_iFKB** | **Dd_iFKC** | **Hs_GCN2** | **Hs_HRI** | **Hs_PERK** | **Hs_PKR** | **Ld_eK2** | **Ld_LdeK** | **Mm_GCN2** | **Mm_HRI** | **Mm_PERK** | **Mm_PKR** | **Pf_IF2K1** | **Pf_IFK2** | **Sc_GCN2** | **Tg_IF2KA** | **Tg_IF2KB** | **Tg_IF2KC** | **Tg_IF2KD** | **Hs_CDK1** |
|  | **Ei_IF2K-A** |  | 48.12 | 64.57 | 43.97 | 29.51 | 25.83 | 25.36 | 27.72 | 31.31 | 28.82 | 29.18 | 29.86 | 32.24 | 17.72 | 30.42 | 29.45 | 30.06 | 31.19 | 28.6 | 13.01 | 32.49 | 17.73 | 7.65 | 17.72 | 27.37 | 13.64 |
|  | **Ei_IF2K-B** | 33.48 |  | 44.96 | 64.88 | 29.81 | 28.96 | 28.41 | 28.62 | 30.02 | 29.33 | 30.63 | 28.57 | 30.69 | 17.86 | 29.41 | 30.15 | 30.76 | 28.83 | 26.77 | 12.18 | 29.09 | 17.82 | 7.12 | 17.12 | 26.09 | 13.36 |
|  | **Eh_IF2K-A** | 49.88 | 31.14 |  | 48.15 | 27.81 | 24.61 | 24.06 | 25.64 | 28.37 | 26.44 | 25.54 | 32.01 | 31.58 | 15.47 | 28.37 | 26.3 | 25.45 | 31.74 | 26.53 | 13.91 | 31.08 | 16.08 | 7.02 | 16.52 | 26.79 | 13.2 |
|  | **Eh_IF2K-B** | 28.12 | 48.39 | 32.46 |  | 28.34 | 27.66 | 27.12 | 28.74 | 27.82 | 29.29 | 29.35 | 28.08 | 29.22 | 16.17 | 27.46 | 29.91 | 30.02 | 28.1 | 25.98 | 12.94 | 29.32 | 18.13 | 7.36 | 17.74 | 25.2 | 13.31 |
|  | **Ac_GCN2-like** | 19.61 | 18.87 | 17.9 | 17.37 |  | 44.76 | 43.92 | 42.14 | 43.86 | 35.67 | 34.41 | 33.18 | 36.69 | 20.33 | 42.71 | 35.05 | 34.54 | 32.25 | 34.97 | 14.12 | 39.15 | 17.3 | 8.19 | 24.89 | 29.85 | 15.43 |
|  | **Dd_iFKA** | 17.27 | 17.6 | 15.26 | 17.03 | 33.11 |  | 98.3 | 42.19 | 36.47 | 29.1 | 34.74 | 24.41 | 29.71 | 19.18 | 34.53 | 28.62 | 35.17 | 23.32 | 26.09 | 11.97 | 36.06 | 20.93 | 9.14 | 22.77 | 22.7 | 12.17 |
|  | **Dd_iFKB** | 16.64 | 17.5 | 14.84 | 17.08 | 32.77 | 97.96 |  | 41.69 | 35.31 | 28.55 | 33.93 | 23.78 | 29.48 | 18.72 | 33.39 | 28.06 | 34.36 | 22.56 | 25.6 | 12.08 | 35.51 | 20.73 | 9.09 | 22.1 | 22.13 | 11.75 |
|  | **Dd_iFKC** | 17.35 | 16.5 | 15.79 | 18.6 | 28.93 | 29.38 | 29 |  | 40.04 | 31.16 | 33.22 | 25.6 | 30 | 21.31 | 39.43 | 31.16 | 33.67 | 24.58 | 26.44 | 13.37 | 40.81 | 17.86 | 8.51 | 23.01 | 24.35 | 13.64 |
|  | **Hs_GCN2** | 18.99 | 18.69 | 18.31 | 18.8 | 31.78 | 25.91 | 25.41 | 28.19 |  | 33.47 | 36.11 | 33.72 | 32.98 | 21.06 | 90.48 | 33.33 | 36.23 | 33.73 | 33.48 | 13.92 | 38.13 | 17.55 | 7.93 | 22.76 | 30.07 | 16.81 |
|  | **Hs_HRI** | 17.56 | 16.57 | 16.86 | 16.6 | 24.74 | 20.58 | 19.84 | 23.12 | 23.59 |  | 37.98 | 33.41 | 31.62 | 20.79 | 32.41 | 88.49 | 38.8 | 34.52 | 30.59 | 15.99 | 35.33 | 17.77 | 7.78 | 18.18 | 29.64 | 15.87 |
|  | **Hs_PERK** | 17.47 | 17.53 | 15.94 | 17.75 | 21.67 | 21.43 | 21.1 | 21.93 | 23.25 | 24.03 |  | 32.16 | 29.79 | 20.39 | 33.89 | 40.12 | 91.79 | 30.6 | 33.79 | 13.76 | 30.38 | 20.59 | 8.09 | 21.95 | 23.42 | 13.97 |
|  | **Hs_PKR** | 21.09 | 18.75 | 21.59 | 19.01 | 23.5 | 17.17 | 16.53 | 18.05 | 23.19 | 21.41 | 23.3 |  | 32.66 | 17.12 | 31.95 | 32.78 | 32.85 | 74.28 | 35.51 | 15.9 | 35.75 | 13.43 | 5.47 | 16.97 | 34.19 | 18.91 |
|  | **Ld_eK2** | 18.07 | 17.07 | 17.89 | 15.69 | 25.16 | 19.81 | 19.71 | 18.25 | 22.06 | 21.37 | 20.11 | 18.84 |  | 17.16 | 32.07 | 33.12 | 28.92 | 31.3 | 33.11 | 13.2 | 34.82 | 16.35 | 6.72 | 18.97 | 30.02 | 14.7 |
|  | **Ld_LdeK** | 9.67 | 9.24 | 8.4 | 7.9 | 11.38 | 10.27 | 10.27 | 12.19 | 11.37 | 11.15 | 11.23 | 9.43 | 9.6 |  | 21 | 20.66 | 21.22 | 17 | 18.04 | 11.93 | 18.83 | 20.5 | 10.91 | 16.7 | 15.16 | 10.25 |
|  | **Mm_GCN2** | 18.89 | 18.24 | 17.66 | 18 | 30.83 | 23.94 | 23.45 | 27.53 | 87.14 | 22.66 | 22.16 | 22.3 | 21.31 | 10.88 |  | 32.27 | 33.83 | 32.48 | 34.62 | 13.49 | 38.41 | 17.31 | 7.74 | 22.98 | 31.15 | 16.81 |
|  | **Mm_HRI** | 17.59 | 16.98 | 16.31 | 17.57 | 24.12 | 21.06 | 20.32 | 22.43 | 23.64 | 82.01 | 25.58 | 21.46 | 21.15 | 11.03 | 22.91 |  | 40.35 | 34.37 | 30.59 | 16.22 | 35.62 | 17.77 | 7.73 | 18.93 | 28.42 | 16.18 |
|  | **Mm_PERK** | 17.81 | 17.86 | 15.52 | 17.18 | 22.2 | 21.72 | 21.39 | 21.89 | 23.58 | 24.71 | 88.71 | 22.79 | 20.23 | 11.71 | 21.75 | 25.87 |  | 30.67 | 33.14 | 14.2 | 30.27 | 20.06 | 8.5 | 22.07 | 23.52 | 14.1 |
|  | **Mm_PKR** | 22.38 | 19.37 | 22.42 | 18.52 | 23.43 | 16.61 | 15.99 | 16.82 | 23.52 | 21.67 | 21.15 | 65.94 | 19.08 | 10.26 | 23.13 | 21.72 | 21.06 |  | 32.1 | 16.77 | 35.71 | 12.87 | 5.47 | 16.36 | 34.88 | 21.53 |
|  | **Pf_IF2K1** | 17.8 | 15.21 | 15.37 | 15.82 | 23.45 | 17.39 | 16.91 | 18.14 | 23.24 | 20.68 | 21.17 | 24.02 | 18.57 | 8.01 | 23.93 | 20.04 | 20.74 | 22.02 |  | 14.82 | 34.95 | 15.33 | 6.83 | 21.56 | 31.6 | 15.63 |
|  | **Pf_IFK2** | 8.61 | 7.63 | 8.8 | 8.31 | 9.47 | 8.64 | 8.72 | 8.92 | 10.02 | 10.61 | 8.96 | 11.51 | 8.14 | 5.49 | 9.61 | 10.81 | 9.26 | 11.68 | 10.54 |  | 15.21 | 8.84 | 7.11 | 10.72 | 14.26 | 8.06 |
|  | **Sc_GCN2** | 22.43 | 18.38 | 18.82 | 19.22 | 30 | 28.05 | 27.3 | 29.6 | 30.28 | 23.77 | 20.58 | 22.75 | 22.54 | 9.87 | 30.04 | 24.25 | 20.11 | 24.23 | 22.86 | 10.73 |  | 16.67 | 7.23 | 20.24 | 33.79 | 17.16 |
|  | **Tg_IF2KA** | 10.19 | 10.5 | 9.39 | 10.38 | 11.29 | 12.95 | 12.85 | 11.09 | 11.21 | 10.64 | 12.35 | 8.21 | 9.49 | 12.16 | 11.02 | 10.53 | 12.46 | 8.09 | 9.18 | 5.36 | 10.51 |  | 15.18 | 15.52 | 12.36 | 6.94 |
|  | **Tg_IF2KB** | 4.09 | 3.82 | 3.35 | 3.97 | 4.96 | 5.55 | 5.6 | 4.87 | 4.99 | 4.39 | 4.75 | 3.74 | 3.85 | 6.39 | 4.87 | 4.54 | 5.06 | 3.63 | 3.65 | 4.34 | 4.35 | 8.98 |  | 6.1 | 5.37 | 3.87 |
|  | **Tg_IF2KC** | 10.54 | 10.41 | 10.38 | 9.92 | 16.29 | 13.98 | 13.58 | 15.63 | 15.78 | 12.84 | 13.88 | 11.53 | 10.77 | 9.72 | 16.29 | 12.72 | 14 | 11.57 | 14.69 | 5.85 | 12.84 | 10.5 | 3.39 |  | 19.32 | 9.05 |
|  | **Tg_IF2KD** | 13.58 | 14.29 | 14.06 | 13 | 18.58 | 13.17 | 13.06 | 13.82 | 19.39 | 19.19 | 13.57 | 20.8 | 17.32 | 7.77 | 20.04 | 18.16 | 13.52 | 19.77 | 18.52 | 7.22 | 19.77 | 6.44 | 2.89 | 11.69 |  | 18.32 |
|  | **Hs_CDK1** | 8.47 | 7.66 | 8.01 | 7.22 | 8.85 | 7.7 | 7.42 | 8.42 | 10.21 | 10.23 | 8.64 | 12.61 | 8.91 | 5.45 | 9.87 | 10.71 | 8.61 | 14.16 | 9.89 | 4.34 | 9.93 | 4.02 | 2.3 | 5.71 | 8.65 |  |
