# Supplementary figures and images for: Eukaryotic Initiation Factor 2α Kinases Regulate Virulence Functions, Stage Conversion, and the Stress Response in Entamoeba invadens
Source: mSphere. 2022 May 31;7(3):e00131-22. doi: 10.1128/msphere.00131-22 (PMC9241534; doi:10.1128/msphere.00131-22)

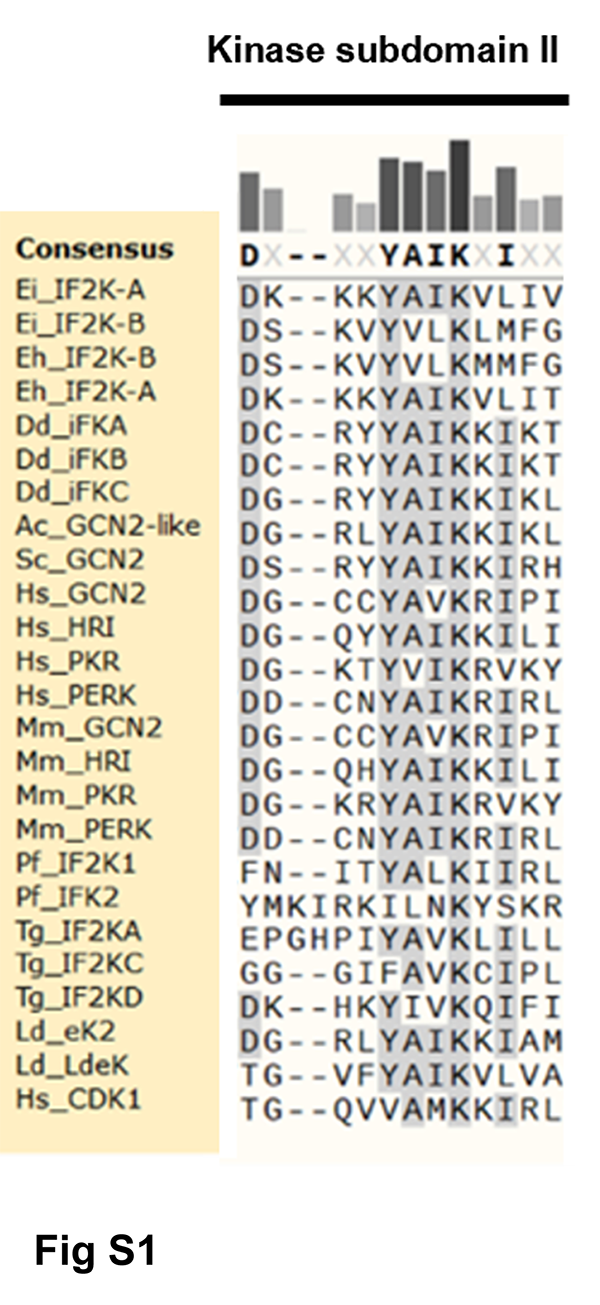

Supplement: FIG S1 [file msphere.00131-22-s0003.tif]

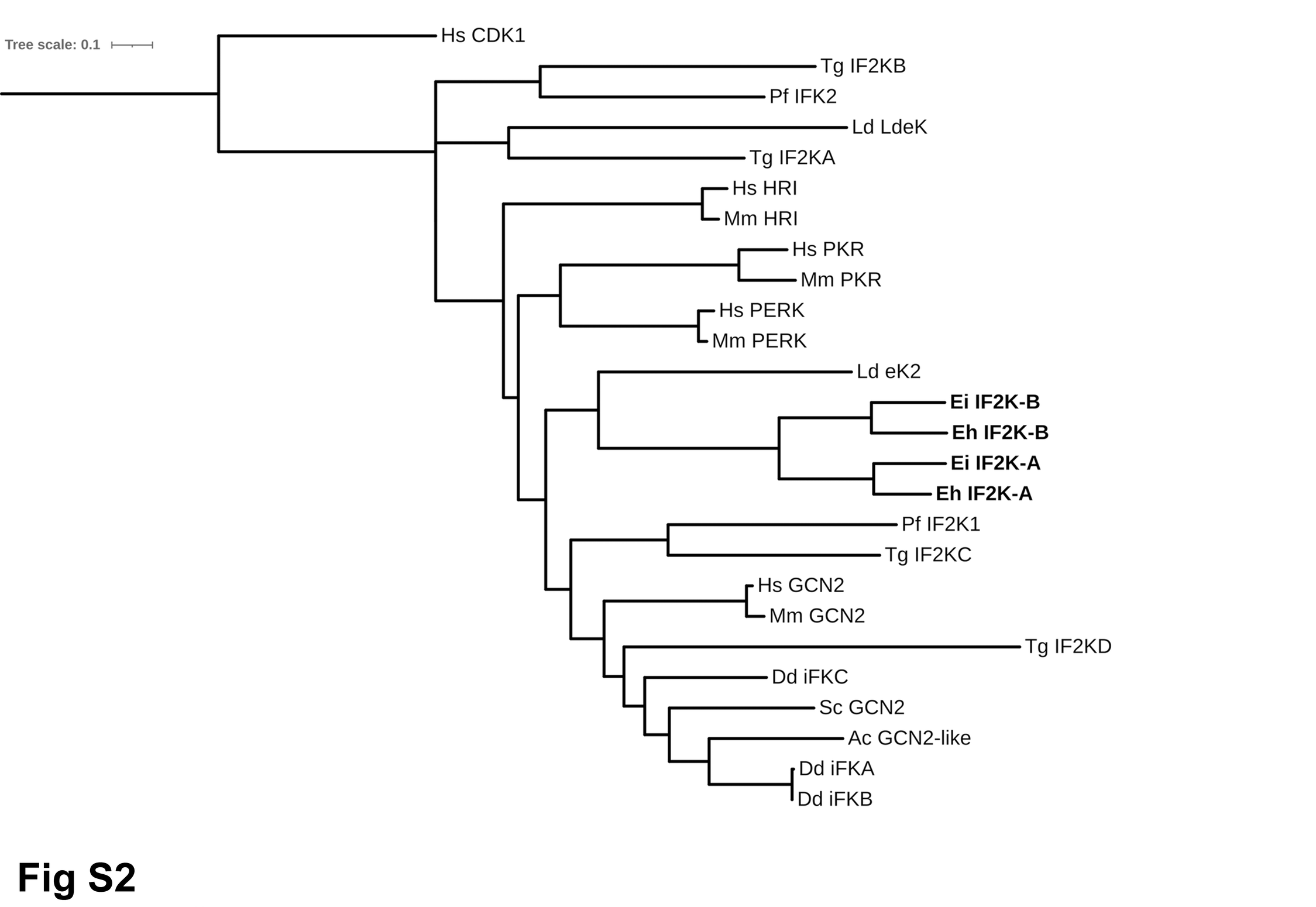

Supplement: FIG S2 [file msphere.00131-22-s0004.tif]

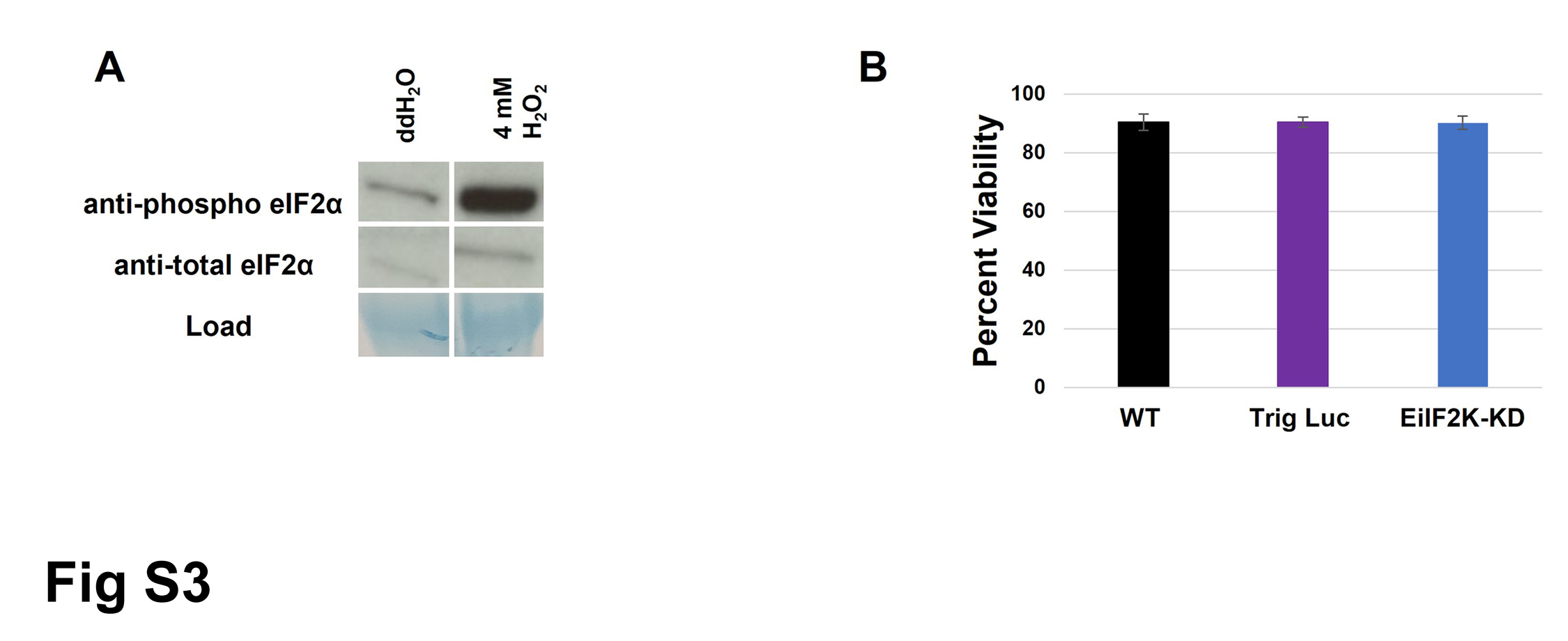

Supplement: FIG S3 [file msphere.00131-22-s0005.tif]
