# Supplementary material for: Eukaryotic Initiation Factor 2α Kinases Regulate Virulence Functions, Stage Conversion, and the Stress Response in Entamoeba invadens
Source: mSphere. 2022 May 31;7(3):e00131-22. doi: 10.1128/msphere.00131-22 (PMC9241534; doi:10.1128/msphere.00131-22)
Supplement: TABLE S2 [file msphere.00131-22-s0002.docx]

| Table S2: Primers used in this study | |
| --- | --- |
| Primer Name | **Sequence** |
| *Cloning Primers* |  |
| EiIF2K-A-AvrII-F (EIN_052050) | 5’-CCCCTAGGATGTCCGTCAC-3’ |
| EiIF2K-A-AvrII-R (EIN_052050) | 5’-CCCCTAGGTTAGTCGGACGGAG-3’ |
| *RT PCR primers* |  |
| EiIF2K-A-F (EIN_052050) | 5’-CGAAGACGAGATGGGTTCTTT-3’ |
| EiIF2K-A-R (EIN_052050) | 5’-CGAAGTGGAGTTCACGATTCT-3’ |
| EiIF2K-B-F (EIN_096010) | 5’-GAAGGCCAACGAGTGAGGAA-3’ |
| EiIF2K-B-R (EIN_096010) | 5’-CTCACTTCTCCGCCACACAT-3’ |
| Trophozoite Internal control-F (EIN_327460) | 5’-CCGACAGCAGAAGAACAAGA-3’ |
| Trophozoite Internal control-R (EIN_327460) | 5’-GGAGATGAGTAAGCGAAGAACA-3’ |
| Cyst Internal Control-F (EIN_162500) | 5’-ACCAGCCGAGGTCAAGAAAG-3’ |
| Cyst Internal Control-R (EIN_162500) | 5’-TCTTCGGGTGTGGCTTTACC-3’ |
| *Mutagenic Primers* |  |
| Dead-EiIF2K-A-F (EIN_052050) | 5’- AGAAGTACGCAATCA**g**GGTATTAATTGTGTC-3’ |
| Dead-EiIF2K-A-R (EIN_052050) | 5’- TCTTATCGTCTTTTCTAATACCACTGTAGAC-3” |
